# Gum Guillotine Forceps

**Published:** 1884-01

**Authors:** L. D. Shepard


*Editor Independent Practitioner :*

In the November issue of your journal, (page 622) is an illustration and description of "Abbott's Scissors," "an instrument devised by Prof. Frank Abbott, for separating the gum on erupting teeth, more especially the third molars, etc."

I bought in London, last summer, a similar instrument, which is made in two sizes and with different curves to adapt it for both upper and lower jaws. I exhibited these instruments at the meeting at Providence, October 7, of the New England Dental Society. Prof. Garretson was present, and was enthusiastic in their praise, declaring that they supplied a want which he had long felt when attempting to remove the opuculum over an erupting tooth. These instruments have been in use in England for two years, and have been advertised in Ash's Catalogue (edition July, 1882), and published to the profession in an editorial in the *British Journal of Dental Science.* You will find in the issue for April 1st, 1882, (page 332) the following :

"GUM GUILLOTINE FORCEPS.

"Messrs. C. Ash & Sons have manufactured a cleverly conceived instrument, the design of Mr. Woodhouse, with which the superincumbent gum over an erupting wisdom tooth can be removed by an instantaneous operation. It resembles a straight, thin, long-bladed pair of forceps, the one blade of which terminates in a thin, flat, shovel-shaped end, somewhat approaching in size the masticating surface of a wisdom tooth ; the opposing blade forms on irregular shaped ring, with a sharp edge, into which the flat blade accurately fits on closing the forceps. In using it an incision has to be made through the gum with a lancet along the anterior margin of the wisdom tooth ; the thin, flat blade of the guillotine forceps can then be inserted and passed over the whole surface of the tooth. With one pinch of the handles the whole surface of the tooth will be exposed, and the piece of gum brought away in the instrument with as much dexterity as a railway porter clips a ticket, and, indeed, much after the same fashion."

The instruments are beautifully made and work to a charm—the only wonder is that such an instrument has not been devised before. The similarity between those of Prof. Abbott and Mr. Woodhouse is very siriking, in fact a description of either would answer for the other. As it is a rule, I think, that credit for an invention or discovery is given to him who first publishes it, unless an account of "Abbott's Scissors" has been published before that in your last issue, this very beautiful and useful instrument should be known as Mr. Woodhouse's, rather than Prof. Abbott's.

Of course any one who knows Dr. Abbott would not for a moment suspect him of introducing as original an instrument the idea of which he had borrowed, especially when it had been so fully and clearly described in so widely circulated and prominent a magazine as the *British Journal.* It is only another of the many illustrations of the independent invention by two or more of the same machine.

It will be good news to the profession that Mr. Woodhouse's Gum Guillotine Forceps will soon be on sale here, as the agent of the S. S. White Dental Manufacturing Co., who was at the meeting in Providence, secured the loan of one of my instrnments to take to Philadelphia as a copy.

I cannot too strongly recommend the instrument to every dentist, and it is only because I consider the invention so meritorious that I make the effort to have the credit rendered to whom it is due.

Very truly yours,

L. D. SHEPARD, D. M. D.

